# Early acupuncture intervention strongly associates with improved swallowing recovery in post-stroke dysphagia: a multicenter real-world cohort study

**DOI:** 10.3389/fneur.2025.1553947

**Published:** 2025-09-15

**Authors:** Junlong Li, Guiping Li, Shu Wang, Li Li, Fei Wang, Chen Yang, Yan Shen, Sha Yang, Zhijie Xu, Fan Xu, Mengnan Lu, Xiaomei Wang, Yong Wang, Zhiliang Zhou, Hao Li, Yongjun Peng, Yue Cao, Xiangfang Chen, Xiaoshan Zheng, Ke Han

**Affiliations:** ^1^The First Teaching Hospital of Tianjin University of Traditional Chinese Medicine, Tianjin, China; ^2^National Clinical Research Center for Chinese Medicine Acupuncture and Moxibustion, Tianjin, China; ^3^Tianjin Key Laboratory of Acupuncture and Moxibustion, Tianjin, China; ^4^The Affiliated Hospital of Tianjin Academy of Traditional Chinese Medicine, Tianjin, China; ^5^Key Laboratory of Cerebropathy Acupuncture Therapy of State Administration of Traditional Chinese Medicine, Tianjin, China; ^6^Beijing University of Chinese Medicine Dongzhimen Hospital Medical in Xiamen, Xiamen, China; ^7^The Second Affiliated Hospital of Tianjin University of Traditional Chinese Medicine, Tianjin, China; ^8^Jiangsu Second Hospital of Traditional Chinese Medicine, Nanjing, China; ^9^Jiangsu Province Hospital of Chinese Medicine, Nanjing, China; ^10^First Affiliated Hospital of Hunan University of Traditional Chinese Medicine, Changsha, China; ^11^Tianjin Wuqing Traditional Chinese Medicine Hospital Affiliated to Tianjin University of Traditional Chinese Medicine, Tianjin, China; ^12^Chifeng Municipal Hospital, Chifeng, China; ^13^Shandong Provincial Hospital Affiliated to Shandong First Medical University Jinan, Jinan, China

**Keywords:** post-stroke dysphagia, acupuncture, timing, swallowing function, real-world study

## Abstract

**Background:**

Post-Stroke Dysphagia (PSD) is a common complication of stroke, significantly impairing recovery and quality of life. Acupuncture has shown potential in improving swallowing function, yet the optimal timing of intervention remains unclear. This study evaluated the effect of acupuncture timing on swallowing recovery in stroke patients.

**Methods:**

We conducted a multicenter real-world study across 27 hospitals in China, enrolling 382 stroke patients with PSD. Inclusion required symptom onset within 90 days and a Water Swallowing Test (WST) score > 2. The primary outcome was swallowing function recovery at discharge, 90 ± 7 days, and 180 ± 7 days post-onset. Multivariate logistic regression was used to adjust for potential confounders.

**Results:**

Among 359 participants with complete data, early acupuncture (0–14 days post-stroke) was strongly associated with improved swallowing recovery. Delayed treatment beyond 28 days significantly reduced recovery odds at discharge (adjusted OR = 2.17; 95% CI: 1.12–4.21) and 90 days (adjusted OR = 2.57; 95% CI: 1.31–5.04). This effect diminished at 180 days (adjusted OR = 1.55; 95% CI: 0.52–4.61). Medullary lesions and diabetes were also associated with poorer outcomes, while hyperlipidemia showed a potential protective effect. The study relied on WST as the sole functional assessment, which, while practical, lacks the sensitivity of instrumental measures like VFSS or FEES for detecting silent aspiration.

**Conclusion:**

Early acupuncture intervention within 2 weeks post-stroke is strongly associated with improved swallowing recovery in real-world clinical practice. Delayed treatment may limit clinical benefit. Future research should incorporate instrumental swallowing assessments and randomized trials to refine acupuncture timing and validate these findings.

**Clinical trial registration:**

ChiCTR2100042721.

## 1 Introduction

### 1.1 Post-stroke dysphagia

Post-Stroke Dysphagia (PSD) is a common complication following an acute stroke, affecting a substantial number of patients. Clinically, PSD significantly increases the risk of aspiration pneumonia, malnutrition, mortality, and other adverse functional outcomes (1). Pathophysiologically, it results from disruptions to a complex network of cortical and subcortical regions involved in swallowing. Immediate screening for PSD is crucial, beginning with simple bedside water-swallowing tests and progressing to more sophisticated assessments using instrumental diagnostics such as flexible endoscopic evaluation of swallowing or videofluoroscopy when indicated. Technological advancements and digitalization in diagnostic procedures are expected to enhance the accuracy of these assessments, paving the way for tailored management strategies based on individual dysfunction patterns and risk factors, which are essential for improving post-stroke quality of life (2).

### 1.2 Assessment and acupuncture treatment of post-stroke dysphagia

Despite recommendations for dysphagia screening protocols for stroke patients, only a few have been validated against gold-standard assessments for aspiration risk (3–5). There is considerable variability across different sites and guidelines concerning the best protocols for dysphagia screening, with performance measures often lacking specificity (6, 7). Moreover, the effectiveness of various swallowing assessments in reducing pneumonia, disability, or death post-stroke remains uncertain (8). For this multicenter study, the Water Swallowing Test (WST) was chosen for its applicability, consistency, economy, and convenience (9).

In clinical practice, simple observational experiments or scales, like the WST, Functional Oral Intake Scale (FOIS), Toronto Bedside Swallowing Screening Test (TOR-BSST), and Gugging Swallowing Screen (GUSS), are frequently used to evaluate swallowing function. The WST, developed by Toshio Kubota, is notable for its straightforward classification and ease of use (9). The specific procedure and evaluation criteria for the WST are as follows: The patient sits upright and drinks 30 milliliters of warm tap water, with the time taken and any coughing observed. Grade 1: Able to swallow the water smoothly in one attempt; Grade 2: Takes more than two attempts but can swallow without coughing; Grade 3: Able to swallow in one attempt, but with coughing; Grade 4: Requires more than two attempts to swallow, with coughing; Grade 5: Frequent coughing, unable to swallow all the water. When the score returns to 2 or below, swallowing function is considered ideally restored, with a low risk of aspiration.

Acupuncture represents a safe and effective alternative therapy for PSD. Existing clinical and experimental evidence suggests that acupuncture can promote neuroplasticity following ischemic stroke, improve neurological deficits, coordinate swallowing muscle movements, and help restore or rebuild the swallowing reflex, thereby enhancing recovery (10, 11).

### 1.3 The importance of real-world evidence in stroke-related research

In recent years, real-world studies (RWS) have garnered increased attention as essential complements to randomized controlled trials (RCTs). These studies adapt interventions to reflect patient preferences and real-world circumstances, thus more accurately reflecting clinical settings and focusing on all treatment outcomes and long-term clinical results. This approach significantly enhances the research findings’ external validity and applicability to everyday practice. Real-world evidence is particularly valuable in assessing treatments like acupuncture’s effectiveness and broad applicability in diverse patient populations (12, 13). Such studies aim to leverage a large array of real-world data to explore the therapeutic effects of acupuncture on swallowing disorders post-stroke, thus providing a theoretical basis and guidance for future clinical research and treatment of these conditions (14, 15).

Recent advancements in acupuncture research highlight its potential role in enhancing standard care practices, especially when traditional interventions are insufficient (16). As acupuncture gains acceptance and evidence supporting its efficacy grows, integrating this modality into conventional treatment protocols for PSD could significantly improve patient outcomes. This integration is supported by extensive research, including clinical searches, systematic reviews, and meta-analyses, confirming acupuncture’s positive impact on neuroplasticity and neurological recovery post-stroke (10, 11). Previous studies have suggested that the peak period of neuroplasticity—namely, the acute phase—is particularly critical for the recovery of swallowing function. Therefore, this study focuses specifically on analyzing the impact of acupuncture timing during the acute phase on the prognosis of PSD in real-world clinical settings.

The development of high-quality, real-world research on acupuncture is crucial for establishing its effectiveness as a treatment modality. Real-world research offers a more comprehensive assessment of how treatments work in varied real-world settings instead of the controlled conditions of RCTs. This type of research not only supports the efficacy found in RCTs but also provides insight into the effectiveness, safety, and patient satisfaction with acupuncture, offering a broader understanding of its benefits and limitations (12).

Large-scale real-world studies and meta-analyses have indicated that acupuncture may reduce mortality and improve recovery outcomes in patients with chronic diseases such as type 2 diabetes, suggesting its potential benefits in stroke rehabilitation (17). These findings emphasize the need for a more comprehensive approach in acupuncture research, utilizing real-world study methods to provide a more complete perspective on acupuncture’s efficacy.

Therefore, our team focused on analyzing the impact of acupuncture as a primary intervention for PSD in real-world medical settings. We considered multiple factors during routine admission, treatment, and post-discharge phases, investigating how the presence or absence of medullary lesions influences recovery from dysphagia under the comprehensive treatment framework with acupuncture as the main intervention.

## 2 Materials and methods

### 2.1 Participants

This study is based on a prospective stroke cohort from the National Center for Clinical Research in Acupuncture and Moxibustion in China, including patients diagnosed with their first-ever cerebral infarction presenting with symptoms of neurogenic dysphagia. Eligibility criteria comprised an onset of symptoms within 90 days, age over 35 years, clinically assessed dysphagia, a WST score above 2, and willingness to undergo acupuncture treatment. Participants were diagnosed and screened across 27 hospitals in 13 provincial regions of China from December 2020 to May 2023. The initial stroke occurrence and presence of medullary lesions were confirmed via imaging studies (CT/MRI). Exclusion criteria encompassed pre-existing dysphagia before the current stroke event, severe cognitive impairment (MMSE score ≤ 10), and inability to cooperate due to blindness, deafness, or consciousness disorders. The study was approved by the institutional review board, and written informed consent was obtained from all participants or their legal guardians.

### 2.2 Functional assessment

The primary objective of this study was to investigate the impact of factors, mainly the timing of acupuncture intervention, on the recovery of swallowing disorders following cerebral infarction. All participants were actual stroke patients attending the participating centers. Consequently, the treatment and management of stroke and its comorbidities and complications were conducted according to clinical guidelines (18, 19). Manual acupuncture is primarily based on the basic treatment protocols for stroke and PSD as recommended by the guidelines (20, 21), and it standardizes the selection of acupoints and the technical operations of acupuncture according to current standard documentation (22). According to these recommendations, the formulation of manual acupuncture protocols follows three key principles: selecting acupoints based on the affected region, the relevant meridians, and syndrome differentiation. Commonly selected acupoints include Shuigou (DU26), Lianquan (RN23), Jinjin and Yuye (EX-HN12), the posterior pharyngeal wall, Fengchi (GB20), Yifeng (SJ17), Lieque (LU7), Zhaohai (KI6), and Tongli (HT5). Ultimately, based on the acupuncture protocols reported by each participating center for PSD, Fengchi (GB20) and Lianquan (RN23) were used as the primary points. The needling technique for Fengchi involved slow insertion at a depth of approximately 5–9 cm toward the laryngeal prominence, aiming to induce sensations of numbness or soreness in the pharyngeal region. For Lianquan, the needle was inserted 1.5–2.5 cm toward the root of the tongue, producing numbness in that area. Acupuncture was administered once daily, with each session lasting 30 min.

A significant characteristic of traditional Chinese medicine in the treatment of chronic diseases is the need to adjust the patient’s treatment prescription after the formulation of an overall plan, taking into account the patient’s constitution, disease progression, geographical location, and the overall state of the climatic environment. Therefore, we did not interfere with minor adjustments made by acupuncturists to the patient’s prescription, provided that these modifications were confined to acupoint selections recommended by clinical guidelines and did not deviate from the core therapeutic intent. Prior to the study’s commencement, all participating centers received uniform training on diagnostic and treatment assessments and passed a consistency evaluation to ensure uniformity in screening criteria and treatment approaches.

The main outcome measures were the recovery of swallowing function during hospitalization, at 90 ± 7 days and 180 ± 7 days post-onset. A good prognosis was determined when WST scores were ≤2 at these time points. Endpoints included recurrence of cerebrovascular disease or death. Assessments were conducted at the bedside or via telephone follow-ups. To manage participant data effectively, our study utilized the “Acupuncture Treatment for Stroke-related Conditions in Real-World Studies Data Management Platform” established by the National Center for Clinical Research in Acupuncture and Moxibustion. Upon inclusion in the study, detailed records of participants’ demographic information, personal and family medical history, hospitalization data, assessment results, laboratory tests, imaging studies, medication and treatment records, and follow-up information were maintained.

### 2.3 Statistical analysis

The primary outcomes and measurements at each time point were complete. Missingness in routine variables such as BMI did not exceed 15%, and missing data for key variables—such as presence of medullary lesions and comorbidities—did not exceed 5%. Multiple imputation was performed to address missing data using R software version 4.3.0, employing the mice package to create five complete datasets, and results before and after imputation were confirmed to have no significant differences. These datasets were analyzed using the function and pooled using the pool function for further analysis. Initially, 26 potential factors influencing dysphagia were identified through a review of relevant literature and clinical experience. Univariate analyses were then performed on these factors. Quantitative data conforming to a normal distribution were presented as mean ± SD and analyzed using the t-test; non-normally distributed data were presented as median (Q25, Q75) and analyzed using nonparametric tests. Categorical data were presented as counts and percentages and analyzed using chi-square or rank-sum tests. Factors with *p* < 0.1 in univariate screening were entered into a multivariate logistic regression model to adjust for confounders and estimate adjusted odds ratios (ORs) with 95% confidence intervals (CIs), with *p* < 0.05 considered statistically significant.

### 2.4 Ethical approval and trial registration

This study received ethical approval from the Ethics Committee of the National Center for Clinical Research in Acupuncture and Moxibustion and the First Affiliated Hospital of Tianjin University of Traditional Chinese Medicine (Approval No. TYLL2021(K)015). The study protocol was registered with the Chinese Clinical Trials Registry (ChiCTR), registration number ChiCTR2100042721.

## 3 Results

**Figure 1:**
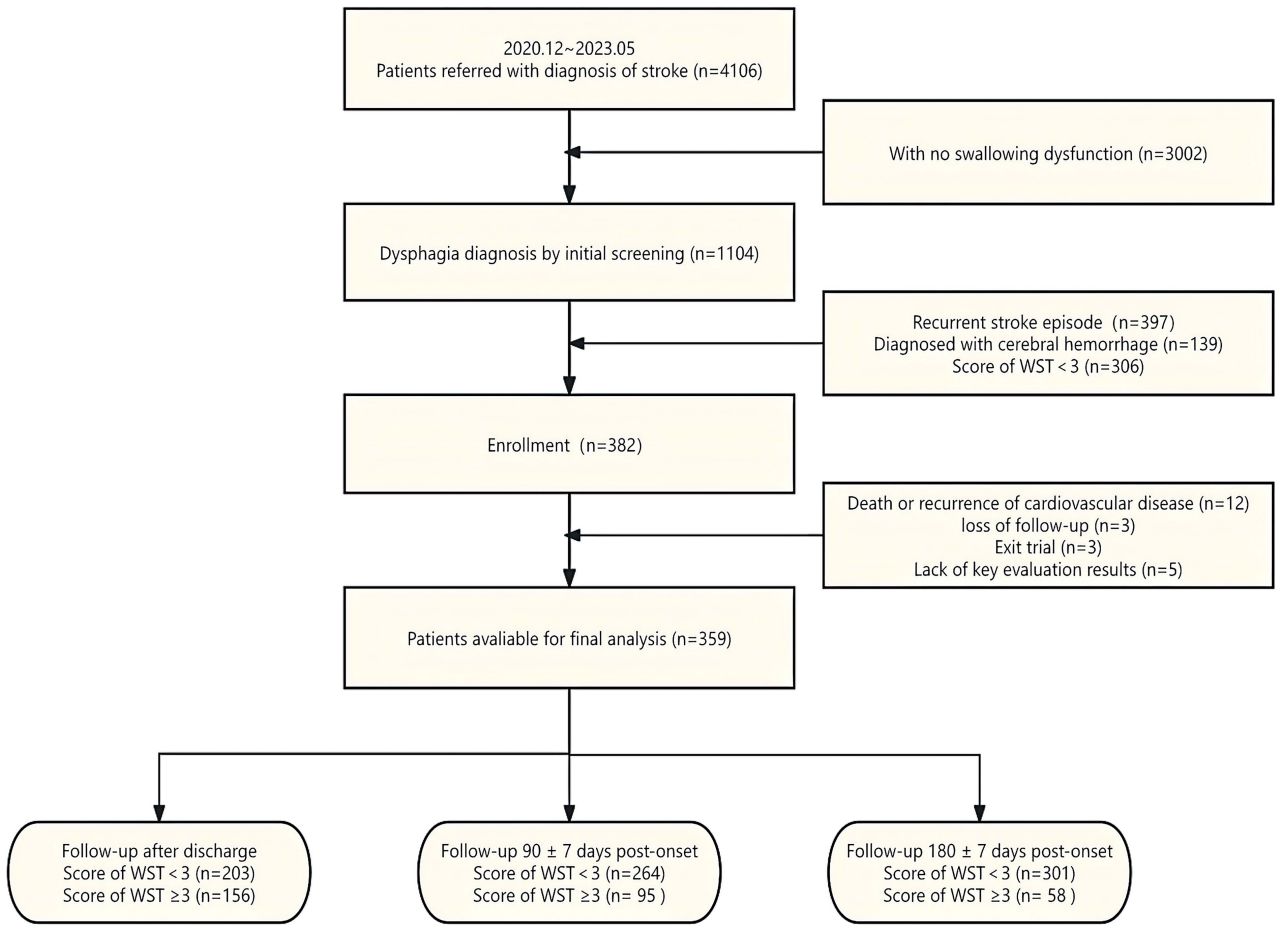
Flow diagram of the trial.

From December 2020 to May 2023, we screened 4,106 patients who visited 27 participating centers for stroke treatment and met the age and onset time criteria for receiving acupuncture treatment. Following the screening process, 382 eligible participants were included in the study. Twelve subjects withdrew from the trial due to death or the onset of cardio-cerebral vascular disease, three subjects withdrew their informed consent during the study, and three subjects were lost to follow-up. After excluding five cases with key missing information, we compiled a dataset with complete data (Figure 1). Detailed records of patients’ ages, genders, BMIs, lifestyles, comorbidities, clinical symptoms, hospitalization, and treatment information were maintained. The timing of acupuncture intervention was stratified into 0–7 days, 8–14 days, 15–21 days, 22–28 days, and over 28 days to explore the impact of acupuncture-related treatment parameters on the recovery of swallowing disorders.

In this study, 359 participants were included. The median age of the participants was 67 years, with 66.02% being male. Most participants (91.64%) were married. Regarding Body Mass Index (BMI), 43.73% had normal weight, 44.01% were overweight, and 9.75% were obese. Concerning lifestyle, 38.16% of participants smoked, and 39.00% consumed alcohol, with 3.34% preferring a bland diet. Additionally, 8.36% had a family history of stroke. In psychological assessments, over 80% of participants reported varying degrees of anxiety or depression. Comorbidities included hypertension (71.31%), diabetes (38.44%), hyperlipidemia (18.94%), coronary artery disease (16.43%), and hyperhomocysteinemia (8.08%). Furthermore, 17.27% had stroke-associated pneumonia. During the treatment phase, 15.04% received thrombolytic therapy, and 11.14% underwent endovascular treatment. The median time to acupuncture intervention was 18 days, with only 16.16% receiving acupuncture within the first 7 days after onset. The use of Traditional Chinese Medicine (TCM) was reported by 65.18% of participants, and the average length of hospital stay was 18 days. These demographic characteristics provide a basic profile of the participants (Table 1).

**Table 1:** Demographic characteristics of participants with post-stroke dysphagia.

Characteristics	Total (*n* = 359)
	M (Q₁, Q₃)/*N*(%)
Age, years	67.00 (59.00, 74.00)
Gender, male	237 (66.02)
Marriage, Married	329 (91.64)
BMI, kg/m^2^	24.22 (22.41, 25.96)
Underweight	9 (2.51)
Normal weight	157 (43.73)
Overweight	158 (44.01)
Obesity	35 (9.75)
Smoke	137 (38.16)
Drink	140 (39.00)
Diet bland	12 (3.34)
Family history of stroke	30 (8.36)
Score of Anxiety/Depression Test	
1 = Not anxious or depressed	70 (19.50)
2 = Slightly anxious or depressed	117 (32.59)
3 = Moderately anxious or depressed	95 (26.46)
4 = Severely anxious or depressed	48 (13.37)
5 = Extremely anxious or depressed	29 (8.08)
Insomnia	87 (24.23)
Irritability	94 (26.18)
Score of NIHSS at admission	8.00 (6.00, 13.00)
Score of WST at admission	
3	192 (53.48)
4	94 (26.18)
5	73 (20.33)
Presence of medullary lesions	32 (8.91)
Abnormal pharyngeal reflex	163 (45.40)
Hypertensive	256 (71.31)
Diabetes	138 (38.44)
Hyperlipemia	68 (18.94)
Coronary artery disease	59 (16.43)
Hyperhomocysteinemia	29 (8.08)
Stroke-associated pneumonia	62 (17.27)
Received thrombolytic therapy	54 (15.04)
Received endovascular treatment	40 (11.14)
Time to acupuncture treatment intervention, days	18.00 (11.00, 34.00)
0–7	58 (16.16)
8–14	81 (22.56)
15–21	74 (20.61)
22–28	28 (7.80)
≥29	118 (32.87)
TCM intake	234 (65.18)
Length of stay	18.00 (13.00, 27.50)

M, Median; Q₁, 1st Quartile; Q₃: 3rd Quartile.

At discharge, and at 90 ± 7 days and 180 ± 7 days post-onset, 43.6, 26.5, and 16.2% of patients, respectively, still experienced poor recovery of swallowing function. Following univariate regression analysis with factors selected for *p* < 0.1, multivariate regression analysis was conducted to explore potential confounding effects and adjust for factors influencing the prognosis of swallowing disorders.

At discharge (Table 2), the univariate analysis indicated that delays between onset and acupuncture intervention were negatively correlated with recovery outcomes, with significant effects observed for interventions beyond 3 weeks (OR = 2.56; 95% CI: 1.01–6.49) and particularly after 4 weeks (OR = 2.73; 95% CI: 1.40–5.29). Patients with medullary lesions also had a significantly lower probability of recovering swallowing function (OR = 2.34; 95% CI: 1.11–4.95). Multivariate analysis adjusting for confounders confirmed the significance of prolonged time to acupuncture intervention and the presence of medullary lesions as predictors of poorer prognosis, with adjusted ORs of 2.17 and 2.32, respectively. Hyperlipidemia was significantly associated with better recovery odds (OR = 0.49; 95% CI: 0.27–0.91).

**Table 2:** Univariable and multivariable analyses of recovery of swallowing disorder—at discharge.

Characteristics	Univariable analysis		Multivariable analysis	
	*p*	OR (95%CI)	*p*	OR (95%CI)
Time to acupuncture treatment intervention, days				
0–7		1.00		1.00
8–14	0.558	1.24 (0.60~2.54)	0.727	1.14 (0.54~2.42)
15–21	0.332	1.43 (0.69~2.96)	0.844	1.08 (0.50~2.34)
22–28	0.047	2.56 (1.01~6.49)	0.118	2.18 (0.82~5.79)
≥29	0.003	2.73 (1.40~5.29)	0.032	2.17 (1.07~4.39)
Score of NIHSS at admission	0.018	1.05 (1.01~1.09)	0.292	1.02 (0.98~1.07)
Score of WST at admission				
3		1.00		1.00
4	0.014	1.88 (1.14~3.11)	0.021	1.88 (1.10~3.20)
5	<0.001	3.93 (2.22~6.95)	<0.001	3.17 (1.71~5.87)
Presence of medullary lesions	0.026	2.34 (1.11~4.95)	0.044	2.32 (1.02~5.27)
Hyperlipemia	0.022	0.52 (0.29~0.91)	0.023	0.49 (0.27~0.91)
Length of stay	0.022	1.01 (1.01~1.02)	0.079	1.01 (1.00~1.02)

OR greater than one indicates greater odds of poor recovery of swallowing disorder. OR, Odds Ratio; CI, Confidence Interval.

At the 90 ± 7 day follow-up (Table 3), univariate analysis showed that longer delays in receiving acupuncture, especially interventions after 3 weeks, continued to impact recovery negatively (OR = 2.89; 95% CI: 1.13–7.37), with a notable effect after 4 weeks (OR = 4.33; 95% CI: 1.81–10.38). Multivariate analysis revealed that medullary lesions and delayed intervention beyond 28 days had adjusted ORs of 2.37 and 2.57, respectively. Diabetes emerged as a significant negative predictor (adjusted OR = 2.66; 95% CI: 1.53–4.64), while hyperlipidemia continued to show a protective effect (adjusted OR = 0.33; 95% CI: 0.15–0.75).

**Table 3:** Univariable and multivariable analyses of recovery of swallowing disorder—at 90 ± 7 days after onset.

Characteristics	Univariable analysis		Multivariable analysis	
	*p*	OR (95%CI)	*p*	OR (95%CI)
Time to acupuncture treatment intervention, days				
0–7		1.00		1.00
8–14	0.308	1.66 (0.63~4.36)	0.732	1.20 (0.43~3.35)
15–21	0.027	2.89 (1.13~7.37)	0.277	1.75 (0.64~4.81)
22–28	0.066	2.91 (0.93~9.10)	0.210	2.17 (0.65~7.29)
≥29	0.001	4.33 (1.81~10.38)	0.050	2.57 (1.00~6.62)
Irritability	0.098	0.62 (0.35~1.09)	0.283	0.71 (0.37~1.33)
Score of NIHSS at admission	0.001	1.07 (1.03~1.12)	0.064	1.05 (1.00~1.10)
Score of WST at admission				
3		1.00		1.00
4	0.026	1.94 (1.08~3.48)	0.062	1.85 (0.97~3.55)
5	<0.001	4.44 (2.45~8.03)	0.001	3.15 (1.57~6.30)
Presence of medullary lesions	0.023	2.36 (1.12~4.96)	0.057	2.37 (0.98~5.75)
Diabetes	0.001	2.22 (1.38~3.58)	<0.001	2.66 (1.53~4.64)
Hyperlipemia	0.017	0.42 (0.20~0.86)	0.008	0.33 (0.15~0.75)
Hyperhomocysteinemia	0.052	0.30 (0.09~1.01)	0.390	0.56 (0.15~2.12)
Received endovascular treatment	0.042	2.03 (1.02~4.00)	0.429	1.38 (0.62~3.08)
Length of stay	0.029	1.01 (1.01~1.02)	0.078	1.01 (1.00~1.01)

OR greater than one indicates greater odds of poor recovery of swallowing disorder. OR, Odds Ratio; CI, Confidence Interval.

By the 180 ± 7 day follow-up (Table 4), the time to acupuncture intervention remained a critical factor, with treatments delayed more than 4 weeks showing a significant negative correlation with recovery (OR = 3.30; 95% CI: 1.20–9.05). Multivariate analysis confirmed this pattern, although the effect size was slightly diminished (adjusted OR = 1.55; 95% CI: 0.52–4.61).

**Table 4:** Univariable and multivariable analyses of recovery of swallowing disorder—at 180 ± 7 days after onset.

Characteristics	Univariable analysis		Multivariable analysis	
	*p*	OR (95%CI)	*p*	OR (95%CI)
Time to acupuncture treatment intervention, days				
0–7		1.00		1.00
8–14	0.487	1.49 (0.48~4.62)	0.973	1.02 (0.31~3.38)
15–21	0.383	1.66 (0.53~5.14)	0.858	1.12 (0.33~3.75)
22–28	0.219	2.30 (0.61~8.73)	0.394	1.86 (0.44~7.81)
≥29	0.021	3.30 (1.20~9.05)	0.432	1.55 (0.52~4.61)
BMI, kg/m^2^				
Normal weight		1.00		1.00
Underweight	0.986	0.00 (0.00~Inf)	0.986	0.00 (0.00~Inf)
Overweight	0.089	0.60 (0.34~1.08)	0.133	0.62 (0.33~1.16)
Obesity	0.035	0.11 (0.01~0.86)	0.045	0.12 (0.01~0.95)
Score of NIHSS at admission	0.012	1.06 (1.01~1.12)	0.233	1.03 (0.98~1.09)
Score of WST at admission				
3		1.00		1.00
4	0.289	1.47 (0.72~2.98)	0.339	1.45 (0.68~3.09)
5	<0.001	3.12 (1.59~6.12)	0.042	2.20 (1.03~4.69)
Presence of medullary lesions	0.059	2.22 (0.97~5.08)	0.108	2.18 (0.84~5.63)
Diabetes	0.050	1.76 (1.01~3.10)	0.090	1.70 (0.92~3.15)
Coronary artery disease	0.087	1.81 (0.92~3.57)	0.114	1.82 (0.87~3.84)
Length of stay	0.043	1.01 (1.01~1.02)	0.042	1.01 (1.01~1.02)

OR greater than one indicates greater odds of poor recovery of swallowing disorder. OR, Odds Ratio; CI, Confidence Interval.

These results suggest that the presence of medullary lesions and delayed acupuncture treatment are important predictors of poor recovery of swallowing function in patients after stroke. Conversely, hyperlipidemia and obesity may have a protective effect on the recovery of swallowing function. These findings enhance our understanding of the factors influencing the recovery of swallowing function after stroke and may guide clinical treatment decisions, providing references for related research on acupuncture treatment prescriptions for post-stroke swallowing disorders.

## 4 Discussion

The clinical efficacy of acupuncture for PSD has been supported by randomized controlled trials (RCTs). However, these studies often do not sufficiently address the influence of variations in acupuncture intervention timing on the recovery of swallowing function under real-world clinical conditions, particularly in the context of integrated Chinese and Western medicine (23, 24). Research conducted in real-world settings offers insights that are more reflective of clinical practice. Our study incorporates acupuncture-related factors such as intervention timing and the concurrent use of Chinese herbal or patent medicines—elements of great clinical interest but relatively understudied (25, 26).

The findings indicate that initiating treatment within 2 weeks post-stroke is likely associated with improved prognostic outcomes, whereas delays beyond 2 weeks may correlate with an increased risk of poor recovery. Notably, treatment initiated more than 4 weeks after stroke appears to be significantly linked to adverse outcomes. Early intervention, particularly within the first 90 ± 7 days post-stroke, shows protective effects that gradually diminish when treatment is delayed to the 180 ± 7 day period. This pattern may reflect the heightened neuroplasticity of the brain during the first 3 months after stroke, a critical window when functional recovery tends to occur (27–29). Beyond 90 days, the brain’s capacity for neuroplastic adaptation declines, potentially explaining the reduced efficacy of interventions initiated later in the recovery process. Consequently, the relative benefits of acupuncture treatment diminish as recovery progresses, and early treated patients may reach a plateau in recovery sooner, resulting in less pronounced differences at the 180-day mark when compared to those with delayed treatment (30). We propose that early acupuncture intervention during this critical recovery window can leverage the natural trajectory of functional restoration, enabling patients to regain independent oral intake sooner and thereby mitigate risks of aspiration pneumonia, dehydration, and malnutrition. Furthermore, by engaging residual brainstem and cortical networks before maladaptive compensatory patterns solidify, early treatment may preserve essential swallowing circuits and prevent long-term synkinetic dysfunction. The attenuation of the negative impact by 180 days suggests two complementary phenomena. First, the first 3 months post-stroke represent a peak period of synaptic sprouting and cortical reorganization; interventions initiated later may not sufficiently augment these intrinsic processes. Second, as patients gradually reach individual recovery ceilings, between group differences are “diluted” over time. Clinically, this indicates that while late acupuncture can still support maintenance of swallowing function, its incremental benefit is smaller underscoring the importance of front loading therapy when plasticity potential is highest.

These findings underscore the potential importance of early acupuncture intervention in managing PSD, as supported by prior research (31–33). Current research generally posits that the recovery from early dysphagia typically occurs within 90 days post-onset, while studies observing persistent abnormal swallowing status after stroke predominantly select a period of 180 days post-onset (34, 35). Consequently, in addition to the observation points at the time of stroke onset and at discharge, we have selected two additional observation time points, namely 90 ± 7 days and 180 ± 7 days post-onset. Patients with medullary lesions who receive acupuncture treatment for dysphagia may have greater difficulties achieving optimal recovery during hospitalization and within 3 months of onset. The median hospital stay was 18 days (IQR: 13–27.5), suggesting that for those discharged home, particularly in the first 3 months post-discharge, the presence of medullary lesions may lead to prolonged feeding tube dependency and increased nursing challenges due to persistent swallowing difficulties, heightening the risk of severe complications such as aspiration pneumonia, dehydration, and malnutrition (36). Outcomes beyond 3 months do not seem significantly correlated with the presence of medullary lesions (34). From a neuroanatomical perspective, the medulla oblongata contains the nucleus tractus solitarius and nucleus ambiguus, which together coordinate both sensory afferents and motor efferents essential for safe swallowing. Lesions in this region disrupt the integrity of these brainstem nuclei and their output pathways, directly impairing pharyngeal-laryngeal muscle coordination and reducing the capacity for neuromodulatory effects induced by acupuncture. Consequently, patients with medullary damage may derive less benefit from acupuncture’s enhancement of brainstem excitability and synaptic plasticity, resulting in the observed poorer outcomes. Once typical symptoms of dysphagia, acupuncturists may perform dysphagia-related acupuncture, although this may be delayed. Predicting the recovery trajectory of PSD is crucial for effective patient management (37), and the location of the brain lesion may be a significant factor influencing the prognosis of acupuncture treatment for PSD (38).

The observation that obesity and hyperlipidemia may have a protective effect on swallowing function recovery at certain time points aligns with findings from previous research. Studies have shown that overweight or obese individuals often experience better functional recovery from chronic diseases such as stroke (39, 40) and tend to have longer survival times (41, 42), a phenomenon referred to as the “obesity paradox” Additionally, evidence points to body weight changes during the treatment and recovery period being more critical to recovery outcomes than BMI at admission (43).

In our analysis, BMI at admission and hyperlipidemia diagnosis were introduced as influencing factors, suggesting that standardized management of blood lipid levels could contribute to improved recovery from dysphagia. However, the apparent protective effect of obesity was significant at only a single follow up time point, and we did not collect direct measures of nutritional status, lean muscle mass, or inflammatory biomarkers, leaving open the possibility of residual confounding. Accordingly, these observational findings should be interpreted with caution rather than viewed as evidence of causality. Future studies incorporating detailed body composition assessments and metabolic or inflammatory markers, as well as longitudinal tracking of weight change, will be essential to clarify the mechanisms underlying this paradox and confirm its clinical relevance in PSD management.

Neurological damage causing PSD is primarily associated with lesions in the brainstem swallowing center and cortical or subcortical structures (2). Acupuncture treatment for dysphagia typically adopts a symptom-focused approach. While Traditional Chinese Medicine (TCM) theory guides acupoint selection, clinicians also evaluate the specific stages of the swallowing process affected, adapting techniques to target regions such as the posterior pharyngeal wall, the base of the tongue, and the uvula area. Our findings align with prior research showing that approximately 15% of dysphagia cases are due to pontine and medullary damage. This type of damage is often more severe than cortical or basal ganglia lesions, with a higher reliance on tube feeding (38). These results suggest that lesion location may significantly influence the efficacy of acupuncture and warrant further exploration into whether treatment protocols should be adjusted based on lesion characteristics, including acupoint selection, needling depth, frequency, and manual techniques.

Our study also reflects the overall therapeutic impact of acupuncture. While efforts were made to ensure sample integrity, baseline characteristics of the 18 withdrawn cases did not differ significantly from those included in the final analysis, although the potential for dropout-related bias cannot be entirely ruled out. However, future research should aim to capture real-world clinical variability more comprehensively. For instance, differences in patient positioning during treatment—such as lateral decubitus for those with severe limb dysfunction versus seated positions for more mobile patients—should be systematically recorded and analyzed. Additionally, refining key technical parameters, such as acupoint selection, manipulation techniques, timing of intervention, and treatment frequency based on individual patient constitutions, could enhance the differentiation of acupuncture approaches. This would enable researchers to fully utilize real-world study designs and provide deeper insights into the nuanced effects of these therapies.

In this multicenter real-world study, the timing and presence of PEG tube placement were inconsistently recorded across sites, and therefore not included in the final analysis. Similarly, while some centers captured data on speech-language therapy participation, limited access to formal rehabilitation services at certain centers precluded uniform analysis. Aphasia and dysarthria, though clinically relevant, were not systematically assessed due to the complexity of their diagnosis and heterogeneity of etiologies. These factors should be incorporated in future studies to improve the comprehensiveness of prognostic modeling.

This multicenter real-world study also has inherent limitations. All WST assessments were performed independently by evaluators with over 5 years of clinical experience; however, the WST’ s sensitivity for detecting silent aspiration and its specificity in grading dysphagia severity are inferior to those of VFSS or FEES, potentially underestimating persistent swallowing dysfunction. Moreover, subjective scoring can introduce inter-rater variability and bias in outcome classification. To address these issues, we plan to incorporate instrumented swallowing evaluations in future work, which will allow more precise detection of aspiration events and clearer visualization of pharyngeal structural abnormalities. There is also variability in equipment and staffing across centers. To ensure consistency in operations and assessments, visual dysphagia indicators such as VFSS or FEES were not used in the present study. In consideration of patient safety, cessation of tube feeding typically occurs after a definite recovery of swallowing function, and following recovery, patients and their families often prefer to extend the duration of acupuncture treatment to consolidate its effects. Although we have accurately recorded the recovery of neurological and swallowing functions, the assessment of dysphagia would benefit from a more detailed schedule of review, such as incorporating additional visits on a monthly or even weekly basis, to determine a more specific recovery timeline.

## 5 Conclusion

Initiating acupuncture treatment early in the course of the disease is advisable. We recommend acupuncture treatment as early as possible, within 2 weeks of onset. The results demonstrate that in the real-world clinical setting of integrated Chinese and Western medicine, early acupuncture intervention significantly enhances recovery of swallowing function. Delays in treatment beyond 3 weeks are associated with poorer outcomes, with significant adverse effects observed at discharge and 90 ± 7 days. However, the impact of delayed intervention beyond 4 weeks diminishes in significance by the 180 ± 7 day follow-up.

Patients with PSD, particularly those with medullary lesions, may face significant challenges in achieving optimal swallowing function during hospitalization and within 3 months of onset, with a higher risk of aspiration. The presence of medullary lesions is a significant predictor of poor recovery at discharge and 90 ± 7 days but shows reduced significance by 180 ± 7 days.

Hyperlipidemia appears to have a protective effect on the recovery of swallowing function at discharge and 90 ± 7 days but not at the 180 ± 7 day follow-up. Similarly, obesity shows a protective effect at the 180 ± 7 day follow-up.

These findings enhance our understanding of the factors influencing the recovery of swallowing function after stroke and may guide clinical treatment decisions. Adjusting acupuncture prescriptions based on lesion location and the timing of intervention could be a future direction for research into acupuncture treatment of PSD. Further studies are needed to refine the intervention time of acupuncture to maximize clinical benefits and to understand the long-term effects of these interventions.

## Data Availability

The datasets presented in this article are not readily available. Requests to access the datasets should be directed to JL, 13612131993@163.com.
